# Ambulatory blood pressure monitoring-based analysis of long-term outcomes for kidney disease progression

**DOI:** 10.1038/s41598-019-55732-4

**Published:** 2019-12-17

**Authors:** Tomoharu Ida, Tetsuro Kusaba, Hiroshi Kado, Takuya Taniguchi, Tsuguru Hatta, Satoaki Matoba, Keiichi Tamagaki

**Affiliations:** 1 Department of Nephrology, Graduate School of Medical Science, Kyoto Prefectural University of Medicine, Kyoto, Japan; 2 Department of Nephrology, Omihachiman Community Medical Center, Shiga, Japan; 3 Department of Cardiovascular Medicine, Graduate School of Medical Science, Kyoto Prefectural University of Medicine, Kyoto, Japan

**Keywords:** Outcomes research, Chronic kidney disease, Renal replacement therapy

## Abstract

Non-dipping nocturnal blood pressure (BP) pattern is a predictor of the future decline of renal function; however, it is unclear whether it is still a risk for chronic kidney disease (CKD) patients with normal BP. To solve this question, a retrospective cohort study was conducted, and 1107 CKD patients who underwent ambulatory blood pressure monitoring (ABPM) were enrolled. We divided patients into 4 groups based on their nocturnal BP dipping pattern (dipper or non-dipper) and average 24-hour BP (hypertension or normotension). The cumulative incidence of composite renal outcomes, including a 40% reduction in eGFR, the induction of renal-replacement therapy, or death from renal causes, was analyzed. Overall, 86.1% of participants were non-dippers and 48.2% of them were normotensive. During the median follow-up period of 4.72 years, the incidence of renal composite outcomes was highest in hypertensive non-dipper patients, and was similar between normotensive dipper and non-dipper patients. Multivariate regression analysis revealed that the 24-hour systolic BP, amount of urinary protein, and hemoglobin values were associated with the incidence of renal outcomes. In conclusion, our ABPM-based analysis revealed that a non-dipping BP pattern with normotension does not predict the future incidence of composite renal outcomes in CKD patients.

## Introduction

The number of chronic kidney disease (CKD) patients is increasing worldwide because of the aging society^1^. Epidemiological studies have reported that high blood pressure (BP) is one of the strongest predictors of CKD onset in the general population and of altered kidney function in CKD patients^2–6^. Based on these findings, international guidelines have highlighted that the reducing the BP in CKD patients is essential to prevent the progression of CKD and cardiovascular diseases^7–9^. However, a target BP is still under debate due to the low reliability and reproductivity of conventional clinical BP measurements.

BP measured outside the hospital, including ambulatory BP monitoring (ABPM), was more strongly correlated with the decline in kidney function and the incidence of cardiovascular diseases than that measured at the clinic^10–12^. Furthermore, a recent large scale cohort of 63910 patients demonstrated that ABPM was superior to home BP measurements for predicting mortality^13^. ABPM enables the detection not only of white-coat or masked hypertension, but also BP circadian rhythms^14^. In general, BP decreases during the night, termed the “dipper” pattern; however, some individuals exhibit less dipping in BP during the night, termed as “non-dippers”. Previous reports demonstrated that the non-dipper BP profile pattern, which is common in patients with CKD^12,15–17^ predicted renal damage, including urinary albumin excretion^18^, the doubling of serum creatinine, and end-stage renal disease (ESRD)^19^.

However, due to the small number of interventional clinical studies with ABPM-based BP controls, the target BP in ABPM has not been established. Furthermore, in CKD patients, though the non-dipper BP pattern is regarded as a risk for the decline of kidney function and progression of cardiovascular diseases^20–23^, it is currently unknown whether a non-dipper BP pattern with normotension can predict the future progression of CKD. Our recent observational report demonstrated that the decline in eGFR over 2 years was correlated with the 24-hour average BP, but not its circadian rhythm^24^. However, the observation period in our recent study was insufficient to conclude that the 24-hour average BP, rather than its circadian rhythm, is an important predictor of the future incidence of ESRD. To overcome this limitation, we conducted a retrospective cohort study to elucidate the impact of actual BP values or its circadian rhythm on the long-term incidence of renal outcomes, including a 40% reduction in eGFR, the induction of renal-replacement therapy, or death from renal causes. In the present study, we divided CKD patients into four categories according to ABPM findings, i.e., 24-hour BP values and BP dipping patterns, and then compared the cumulative incidence of composite renal outcomes.

## Methods

### Patients and study protocol

**Figure 1:**
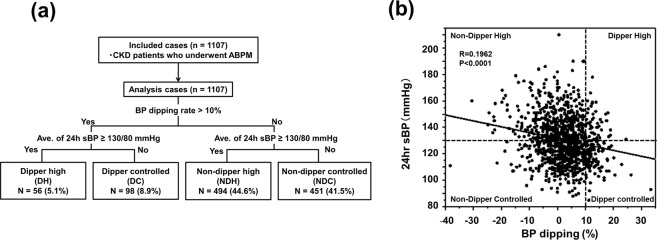
Patient recruitment and distribution of dipper/non-dipper patients with high/controlled BP, and the relationship between the average 24-hour sBP and nocturnal BP dipping rate. (**a**) Flowchart of the process of patient recruitment. A patient with a less than 10% nocturnal decline in systolic BP was defined as a non-dipper. Hypertension was defined as an average 24-hour BP higher than 130/80 mmHg. In total, 86.1% of patients were categorized as non-dippers. (**b**) The average 24-hour sBP correlated with the nocturnal sBP dipping rate (r = 0.1962, p < 0.0001).

We conducted a retrospective cohort study at the Omihachiman Community Medical Center in Shiga Prefecture, Japan. We enrolled 1107 CKD patients who underwent ABPM from October 2006 to July 2016. CKD was diagnosed according to the criteria of the National Kidney Foundation defined as eGFR <60 ml/min/1.73 m^2^ (Fig. 1)^25^. All clinical parameters were obtained from medical records, including age, gender, complications, and laboratory tests, which were performed within 2 weeks of the ABPM procedure. Creatinine (Cr), blood urea nitrogen (BUN), and hemoglobin were measured by a standard procedure. eGFR was calculated according to the following formula^26^: “194 × [age (years)]^−0.287^ × [serum creatinine (mg/dl)]^−1.094^ × [0.739 if female]”. Urine was collected for 24 hours and daily protein excretion was measured. The co-existence of DM was defined as a history of glucose reduction treatment or HbA1c value above 6.5% by the National Glycohemoglobin Standardization Program (NGSP). The body mass index (BMI) was defined as [weight (kg)]/[height (meters)]^2^. The protocol for the present study was approved by the ethics committee of the Omihachiman Community Medical Center (Approval number 28–46). The entire protocol of the present study was designed in accordance with the Declaration of Helsinki. Due to the retrospective design and its low risk to the patients, the ethical committee approved the use of the following opt-out methodology: The requirement for verbal informed consent was waived and informed consent was obtained by generally accessible information as well as easy methods to opt out, which were displayed on the website and at the outpatient clinic of Omihachiman Community Medical Center. Patient data were anonymized and de-identified prior to analysis.

### ABPM procedure and definition of BP profiles

ABPM devices (FB-270, Fukuda Denshi Co., Ltd., Tokyo, Japan) were fit between 15:00 and 16:00 and removed at 15:30 the next day. The cuff size was selected based on the arm circumference and applied to the non-dominant arm. During the night (22:00–07:00), measurements were taken in 1-hour intervals, whereas daytime measurements (07:00–22:00) were taken in 30-minutes intervals. During the ABPM, patient activities were not restricted, but they were asked to stay motionless at the time of measurement. Diurnal and nocturnal BP were defined arbitrarily as 7:00–22:00 and 22:00–7:00, respectively.

The average 24-hour BP was measured by ABPM. Nocturnal BP dipping was quantified as the difference between the average diurnal systolic BP (sBP) and average nocturnal sBP, and expressed as the percentage of average diurnal sBP. The non-dipper pattern was defined as an average diurnal sBP that was not 10% higher than the average nocturnal sBP. Hypertension was defined as an average 24-hour sBP higher than 130/80 mmHg (Fig. 1).

### Outcomes

We analyzed composite renal outcomes, including a 40% reduction in eGFR sustained for at least two consecutive measurements, the induction of renal-replacement therapy (maintenance dialysis or renal transplantation), and death from renal causes (defined as death with a proximate renal cause, typically hyperkalemia), which were used in a recent clinical trial^27^. The date of 40% reduction in eGFR was defined as the latter date of two consecutive measurements, as mentioned above. The date of the induction of renal replacement therapy was the day of the first dialysis session and day of renal transplantation. The dates of major renal outcomes were ascertained from patient records. All major renal outcomes were confirmed by at least two board-certified nephrologists of the Japanese Society of Nephrology.

### Statistical analysis

Data are expressed as the median (interquartile range (IQR)). Comparisons of two groups were performed using Welch’s *t-*test for continuous variables and Fisher’s exact test for categorical variables. Multiple comparisons among the four groups were performed by the Steel-Dwass test for continuous variables and by Bonferroni corrections for categorical variables. We examined the relationship between the average 24-hour sBP and the nocturnal sBP dipping rate using a linear correlation model.

Death before the incidence of composite renal outcomes was considered to be a competing risk event. We compared the cumulative incidence of renal outcomes using Kaplan-Meier curves plotted according to previous recommendations^28^ and by a competing adjusted model. Significant differences among groups were assessed by Wilcoxon’s test.

Cox’s regression analyses were performed in order to assess variables associated with the incidence of composite outcomes using gender, age, the nocturnal sBP dipping rate, and factors considered to have a significant relationship. A P-value less than 0.05 was considered to be significant. Statistical analyses were performed using JMP version 9.0.3 for Windows (SAS Institute Inc., Cary, NC, USA).

## Results

### High prevalence of the non-dipping BP profile in CKD patients

The mean (+SD) age of all participants was 69.2 ± 11.5 years, and 68.9% were men (Table 1). The 1107 patients were divided into the following four groups: non-dipper with high BP (NDH 44.6%), non-dipper with controlled BP (NDC 41.5%), dipper with high BP (DH 5.1%), and dipper with controlled BP (DC 8.9%) (Fig. 1a). The average nocturnal decrease in BP was 2.2 ± 7.7%, and a high prevalence of the non-dipping BP pattern (86.1%) was observed (Fig. 1a, Table 2).

**Table 1:** Patient characteristics of each group for all patients.

	Total	Dipper high	Dipper controlled	Non-dipper high	Non-dipper controlled
Number	1107	56	98	494	459
Men (%)	68.9	66.1	69.4	71.1	66.9
Age (years old)	70 (63–78)	70 (63–77)	71 (64–79)	70 (63–77)	71 (63–78)
BMI (kg/m^2^)	23.30 (21.07–25.86)	23.88 (21.10–27.25)	23.19 (21.17–25.00)	23.90 (21.46–26.29)	23.04 (20.82–25.25)*
Diabetes mellitus (%)	41.0	46.4	28.6 **	49.8	33.3 *
Creatinine (mg/dL)	1.86 (1.39–2.76)	2.16 (1.47–3.21)	1.85 (1.27–2.48)	2.01 (1.46–3.18)	1.69 (1.37–2.36)*
eGFR (ml/min/1.73 m^2^)	29.14 (19.12–40.54)	24.42 (15.43–36.77)	29.32 (20.81–43.52)	26.57 (16.25–37.83)	32.37 (22.46–41.71)*
Blood urea nitrogen (mg/dL)	29.30 (22.10–41.40)	34.75 (24.65–52.80)	28.70 (21.35–36.93)	30.20 (22.30–43.80)	28.00 (21.80–38.75)
Urinary protein (g/day)	0.33 (0.07–1.35)	0.64 (0.10–1.64)	0.17 (0.04–0.40)**	0.90 (0.19–2.90)	0.12 (0.04–0.55)* ***
Hemoglobin (g/dL)	11.30 (9.90–12.70)	11.05 (9.38–12.53)	11.40 (9.83–12.98)	11.05 (9.70–12.50)	11.40 (10.10–12.85)
Calcium channel blockers (%)	70.8	87.5	62.2** ****	79.2	61.7* ***
RAS inhibitors (%)	73.1	71.4	63.3**	77.1	71.0
Diuretics (%)	34.9	37.5	26.5	38.3	32.7
β-blockers (%)	17.8	17.9	15.3	20.7	15.3
Number of antihypertensive drug classes (Ave ± SD)	1.96 ± 1.04	2.14 ± 0.98	1.67 ± 1.07**	2.15 ± 0.99	1.81 ± 1.06*

*p < 0.05 between NDH and NDC, **p < 0.05 between NDH and DC, ***p < 0.05 between NDC and DH, ****p < 0.05 between DC and DH.

BMI: body mass index, RAS: renin angiotensin system, NDH: non-dipper high, NDC: non-dipper controlled, DH: dipper high, DC: dipper controlled.

**Table 2:** Average BP profiles of each group for all patients.

	Total	Dipper high	Dipper controlled	Non-dipper high	Non-dipper controlled
**24-hour (mmHg)**					
Systolic BP	129 (118–141)	140 (133–147)	116 (111–123)*	142 (135–151)^§^	118 (111–124)^† ‡^
Diastolic BP	76 (69–83)	80 (74–86)	72 (66–76)*	81 (76–88)^§^	71 (66–76)^† ‡^
**Daytime (mmHg)**					
Systolic BP	131 (120–143)	149 (142–156)	124 (117–131)^*♯^	142 (135–152) **§	119 (112–125)^† ‡^
Diastolic BP	77 (71–84)	84 (77–92)	76 (71–82)^*♯^	82 (76–90)§	72 (67–78)^† ‡^
**Nighttime (mmHg)**					
Systolic BP	127 (115–141)	128 (122–135)	108 (101–113)^*♯^	142 (135–152) **§	117 (110–123)^† ‡^
Diastolic BP	73 (67–82)	73 (68–79)	64 (59–70)^*♯^	81 (74–88) **§	70 (64–75)^†^
BP dipping (%)	2.8 (−2.4–7.0)	12.8 (11.8–14.3)	12.8 (11.3–15.1)^*♯^	0.0 (−4.3–4.8)^§^	2.4 (1.8–5.8)^† ‡^

*p < 0.05 between NDH and DC, **p < 0.05 between NDH and DH, ^♯^p < 0.05 between NDL and DC, ^†^p < 0.05 between DC and DH.

^‡^p < 0.05 between NDC and DH, ^§^p < 0.05 between NDC and NDH.

BP: blood pressure, NDH: non-dipper high, NDC: non-dipper controlled, DH: dipper high, DC: dipper controlled.

Regarding the clinical characteristics of participants, there was not significant differences in gender age, hemoglobin, or the usage rates of diuretics, or β-blockers among the groups (Table 1). The percentage of patients with DM, serum creatinine levels, eGFR at baseline, amount of urinary protein, and usage of calcium channel blockers significantly differed between NDC and NDH patients.

Based on BP profiles in each group (Table 2), the average 24-hour sBP was similar in NDH and DH patients (142 mmHg vs. 140 mmHg). The average 24-hour sBP was also similar in DC and NDC patients (116 mmHg vs. 118 mmHg). Due to the lack of a decrease in BP during the night in non-dipper patients, the average nocturnal sBP was significantly higher in NDH and NDC patients than in DH and DC patients, respectively. The linear correlation analysis demonstrated a weak but significant relationship between the average 24-hour sBP and BP dipping rate (p < 0.0001, r = 0.1962, Fig. 1b).

### Renal outcomes

**Figure 2:**
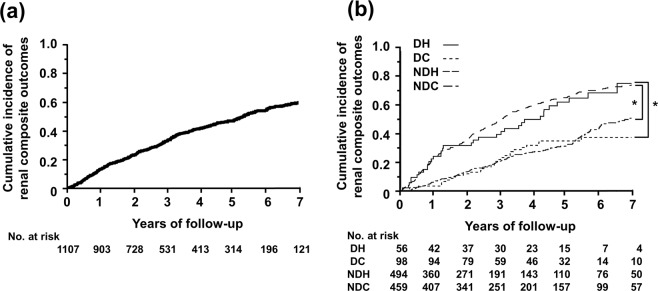
Kaplan-Meier plots of the cumulative incidence of composite renal outcomes in this cohort. (**a**) Among all patients, the cumulative incidence rates of renal events 1, 3, and 7 years after recruitment were 13.5, 32.5, and 58.1%, respectively. (**b**) The cumulative incidence of renal events was greater in patients with hypertension than in those with normotension. *Wilcoxon’s test p < 0.05.

During the follow-up period (median, 4.72 years), 13.5, 32.5, and 46.1% of patients had the composite outcomes of a sustained 40% reduction in eGFR, renal-replacement therapy, or death from renal causes at 1, 3, and 5 years after recruitment, respectively (Fig. 2a). Patients were lost due to the incidence of outcomes, stopping of hospital visits, and death from non-renal causes, and the detailed profiles of annual incidences are summarized in Supplementary Table 1. In a separate analysis according to BP and its circadian rhythm, renal composite outcomes were less frequent among patients with NDC and DC than among those with NDH and DH (Fig. 2b, Supplementary Table 1). The incidence of ESRD was also lower among patients with NDC and DC than among those with NDH and DH (Supplementary Fig. 1a,b, Supplementary Table 2). These results suggest that the average 24-hour sBP value is more important for predicting the future incidence of composite renal outcomes. Of note, in contrast to previous studies reporting the risk of the non-dipping BP profile for the progression of kidney disease^20^, our study suggested that the normotensive non-dipper BP profile is not a risk factor for the incidence of renal outcomes.

**Figure 3:**
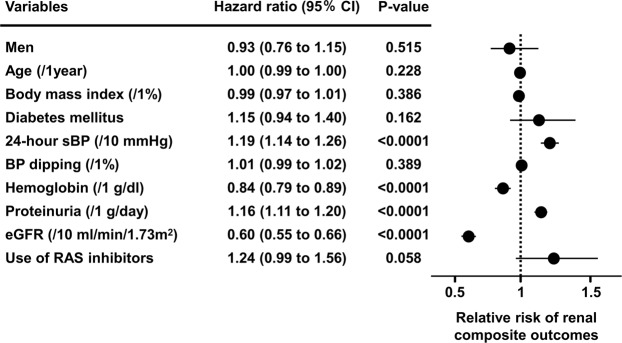
Cox regression analysis for the identification of factors associated with the incidence of renal composite outcomes. Filled circles represent the harzard ratio and horizontal lines denote the 95% confidence intervals (95% CI).

To investigate relevant factors for developing kidney disease, we performed a Cox regression analysis adjusted by gender, age, the nocturnal BP decline rate, and factors considered to have a significant relationship with the incidence of renal composite outcomes. The analysis identified average 24-hour sBP (HR 1.19, 95% CI: 1.14 to 1.26), proteinuria (HR 1.16, 95% CI: 1.11 to 1.20), eGFR at baseline (HR 0.60, 95% CI: 0.55 to 0.66), and hemoglobin values (HR 0.84, 95% CI: 0.79 to 0.89) as significant factors associated with the incidence of renal composite outcomes (Fig. 3). We additionally performed Cox regression analysis of the incidence of ESRD, which resulted in similar results to those for the composite renal outcomes, demonstrating that the average 24-hour sBP (HR 1.28, 95% CI: 1.14 to 1.43), proteinuria (HR 1.31, 95% CI: 1.17 to 1.46), eGFR at baseline (HR 0.38, 95% CI: 0.31 to 0.45), and hemoglobin values (HR 0.85, 95% CI: 0.76 to 0.94) are significant factors associated with the incidence of ESRD (Supplementary Fig. 2).

### BP dipping pattern and the incidence of renal composite outcomes in early and advanced CKD patients

One concern in our analysis was the difference in baseline eGFR among groups, and our multivariate analysis confirmed its strong correlation with the incidence of renal outcomes. In order to exclude the impact of baseline eGFR on the future incidence of renal composite outcomes, we performed a separate analysis on advanced (eGFR < 30) and early (eGFR > 30) CKD patients. In early CKD patients, the baseline eGFR was not significantly different among groups (Supplementary Table 3). In advanced CKD patients, it was the lowest and the amount of urinary protein was the highest in NDH patients (Supplementary Table 4).

Regarding the BP profile in early CKD patients (Supplementary Table 5), the nighttime average sBP was higher in NDH patients than in DH patients (139 mmHg vs. 123 mmHg, p < 0.0001), whereas the average 24-hour sBP was similar (139 mmHg vs. 134 mmHg). Regarding BP profiles in advanced CKD patients (Supplementary Table 6), the nighttime average sBP was higher in NDH patients than in DH patients (144 mmHg vs. 133 mmHg, p < 0.0001), whereas the average 24-hour sBP was similar (144 mmHg vs. 143 mmHg).

**Figure 4:**
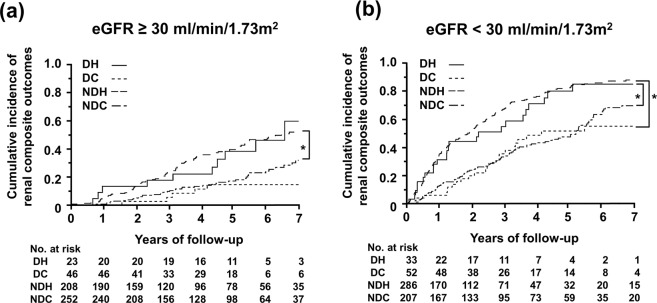
Kaplan-Meier plots of a separate analysis of the cumulative incidence of composite renal outcomes in early and advanced CKD patients. In early (**a)** and advanced (**b**) CKD patients, the cumulative incidence of renal outcomes was greater in patients with hypertension than in those with normotension. *Wilcoxon’s test p < 0.05.

In the analysis of early CKD patients, renal composite outcomes were less frequent among patients with NDC or DC than among those with NDH or DH (Fig. 4a, Supplementary Table 7). Similar to early CKD patients, in the analysis of advanced CKD patients, renal composite outcomes were less frequent among patients with NDC or DC than among those with NDH or DH (Fig. 4b, Supplementary Table 8). Regarding the incidence of ESRD, in both analyses of early and advanced CKD patients, ESRD was less common among patients with NDC or DC than among those with NDH or DH (Supplementary Fig. 3).

## Discussion

Based on previous studies demonstrating the non-dipping BP pattern to increase the risk of developing ESRD^19^, our clinical investigation was performed in order to clarify the impact of the non-dipping pattern of BP on the incidence of composite renal outcomes in normotensive CKD patients. A major result of this retrospective cohort study using ABPM was that the normotensive non-dipper pattern was not a risk for the future incidence of renal composite outcomes. Multivariate analysis demonstrated that a high average 24-hour BP more strongly correlated with the incidence of renal composite outcomes than the BP circadian rhythm.

Although ABPM provides extensive information, including BP circadian rhythms and BP during sleep, which are not monitored by other modalities^12^, the number of large-scale cohorts using ABPM is limited due to its inconvenience for patients. The number of interventional trials conducted to date is also small, and only one study has used ABPM-based BP control for pediatric hypertensive patients^29^. Digital health innovations are emerging in the BP monitoring field by wearable devices using cuffless BP sensors, which facilitate the continuous monitoring of BP^12,30^. The reliability and reproductivity of these products are important for clinical practice^12^, and medical professionals, hypertensive patients, and the general public are interested in these technologies^30^. Therefore, based on the increased demand for clinical evidence of a relationship between long-term outcomes and ABPM, the present study suggested that patients should consider BP values over 24 hours, not its circadian rhythm, in order to prevent the progression of kidney disease.

In contrast to several previous investigations demonstrating the risk of the non-dipper pattern for CKD progression, our study revealed that the non-dipping BP pattern is not associated with the incidence of renal outcomes. One possible explanation for this discrepancy is that most previous analyses were performed by the categorization of dipper vs. non-dipper, not by the BP decline, as continuous variables^19,20,31,32^. The BP of the study group with the non-dipping BP pattern in previous reports was often higher than that of dipping groups^19,20,31,32^, and it may have affected their results. Indeed, the impact of BP on renal outcomes was lost or diminished by adjusting for the BP value in some reports^21,33^ Regarding the impact of normotensive non-dipper pattern of renal outcomes, few studies including our previous one have been reported, but the results were not conclusive^20,24,34,35^. In contrast to our previous study^24^, a normotensive non-dipping BP can predict renal function decline in CKD patients^20,34,35^. However, the number of patients in these studies was small and the outcome was only the short-term renal function decline rate, not the incidence including ESRD. As we used composite renal outcomes, including ESRD and 40% eGFR reduction, in our study, our analysis provides stronger evidence for predicting the future renal prognosis, especially in normotensive non-dipping patients.

However, whether shifting the BP from the non-dipper to dipper pattern by the anti-hypertensive drugs administration has beneficial effects on renal outcomes is uncertain. In order to elucidate the impact of nocturnal BP dipping on renal outcomes, a prospective investigation of shifting the BP pattern by bedtime administration of antihypertensives may be ideal. Bedtime antihypertensive treatment for CKD patients reduced the average nocturnal BP and the percentage of non-dipper patients^36^. In addition, normalizing the BP circadian rhythm by administering anti-hypertensive drugs reduced not only the urinary albumin excretion in diabetic kidney disease^37,38^, but also the incidence of cardiovascular diseases^39–41^. However, in our observational study, the circadian rhythm of the BP profile did not correlate with the future incidence of renal outcomes in both hypertensive and normotensive individuals, suggesting that the benefit of shifting from the non-dipper to dipper pattern to prevent the progression of kidney disease is limited.

The present study has several limitations. We only assessed single-point ABPM at recruitment. Sleep-disrupting cuff inflation during the night may increase the average nocturnal BP. The daily lifestyles of patients, e.g. food intake, may be affected during ABPM in order to obtain a better profile, resulting in a different BP from baseline. Previous studies demonstrated that some patients showed a different BP pattern in serial ABPM, even with short-term intervals^42,43^. In addition, as we obtained no BP measurements at later time points, including ABPM or other methods, the impact of BP control on the future incidence of renal outcomes remains uncertain.

The second limitation was the low prevalence of dipper patients, particularly hypertensive dippers. The small sample size in these populations may have influenced the significance of differences, particularly in the separate analysis according to eGFR. Therefore, the incidence of renal outcomes in DH and DC patients at a later time point needs to be carefully evaluated.

Furthermore, though we revealed that the non-dipping pattern with normotension was not a risk for the incidence of renal outcomes, we did not provide an actual value for the risk threshold for the 24-hour average BP. As this was an observational study on the prognostic value of ABPM, there was no direct intervention regarding the benefits of baseline patient treatments or BP control. In addition, we selected only the average 24-hour sBP, not diurnal or nocturnal average sBP for the analysis; therefore, which BP value among these is most strongly associated with the renal outcomes remains unclear. In order to establish a target BP, more substantial evidence from a large-scale prospective interventional study that includes multiple tests on ABPM is needed. In addition, this was a study on an Asian population at a single center, and a recent nationwide analysis demonstrated variations in ABPM profiles among races and countries^44^. Thus, these results need to be carefully applied to individuals of other races or countries.

The last limitation was the lack of data regarding the office BP and home BP measurement. We were unable to collect information on the situation when the BP was measured from patient records, which is essential for evaluating not only home BP but also office BP.

In conclusion, our ABPM-based analysis demonstrated that a non-dipping BP pattern with normotension does not predict the incidence of renal composite outcomes in CKD patients. These results suggest that the control of BP, rather than its circadian rhythm, is essential to potentially prevent ESRD. A larger scale study to establish the target BP and interventional trials on ABPM-based BP control are required in order to obtain more conclusive evidence.

## Supplementary information

Supplementary tables and figures

## Data Availability

Data are available from the corresponding author upon reasonable request.
