# Supplementary material for: Ambulatory blood pressure monitoring-based analysis of long-term outcomes for kidney disease progression
Source: Sci Rep. 2019 Dec 17;9:19296. doi: 10.1038/s41598-019-55732-4 (PMC6917780; doi:10.1038/s41598-019-55732-4)
Supplement: Supplementary file 1 — Supplementary tables and figures [file 41598_2019_55732_MOESM1_ESM.docx]

Supplementary tables and figures

**Ambulatory blood pressure monitoring-based analysis of long-term outcomes for kidney disease progression**

Tomoharu Ida, Tetsuro Kusaba, Hiroshi Kado, Takuya Taniguchi, Satoaki Matoba, Tsuguru Hatta, and Keiichi Tamagaki

Supplementary Table 1. Detailed profiles of the annual incidence of loss of follow-up, including the incidence of outcomes, stopping of visits, and death from non-renal causes in all patients.

Supplementary Table 2. Detailed profiles of the annual incidence of loss of follow-up, including the incidence of ESRD, stopping of visits, and death from non-renal causes in all patients.

|  | Total | Dipper high | Dipper controlled | Non-dipper high | Non-dipper controlled |
| --- | --- | --- | --- | --- | --- |
| Number | 529 | 23 | 46 | 208 | 252 |
| Men (%) | 70.5 | 60.9 | 80.4 | 68.8 | 71.0 |
| Age (years old) | 70 (63 - 76) | 69 (66 - 75) | 71 (65 - 78) | 69 (63 - 75) | 69 (63 - 77) |
| BMI (kg/m^2^) | 23.55 (21.27 - 26.02) | 23.83 (20.63 - 25.57) | 23.19 (21.68 - 25.00) | 24.12 (21.55 - 26.60) | 23.25 (21.15 - 25.52) |
| Diabetes mellitus (%) | 38.9 | 30.4 | 30.4 | 48.1 | 33.7 * |
| Creatinine (mg/dL) | 1.37 (1.21 - 1.56) | 1.29 (1.19 - 1.65) | 1.27 (1.14 - 1.54) | 1.37 (1.23 - 1.56) | 1.39 (1.22 - 1.55) |
| eGFR (ml/min/1.73m^2^) | 41.12 (35.43 - 47.11) | 42.41 (33.71 - 46.37) | 45.01 (35.67 - 51.48) | 40.51 (34.88 - 47.15) | 40.99 (35.80 - 46.51) |
| Blood urea nitrogen (mg/dL) | 22.40 (18.50 - 27.00) | 23.70 (19.95 - 28.10) | 21.65 (17.73 - 26.28) | 22.05 (18.10 - 26.93) | 22.60 (18.90 - 27.33) |
| Urinary protein (g/day) | 0.20 (0.06 - 0.93) | 0.32 (0.07 - 1.83) | 0.08 (0.01 - 0.25) ** | 0.48 (0.11 - 1.74) | 0.15 (0.05 - 0.56) * |
| Hemoglobin (g/dL) | 12.20 (11.00 - 13.60) | 12.30 (10.80 - 14.00) | 12.15 (10.73 - 13.45) | 12.00 (10.80 - 13.63) | 12.25 (11.10 - 13.60) |
| Calcium channel blockers (%) | 66.2 | 82.6 | 56.5 | 75.5 | 58.7 * |
| RAS inhibitors (%) | 69.4 | 65.2 | 63.0 | 74.5 | 66.7 |
| Diuretics (%) | 28.0 | 26.1 | 23.9 | 29.3 | 27.8 |
| β-blockers (%) | 18.1 | 21.7 | 10.9 | 22.6 | 15.5 |
| Number of antihypertensive drug classes (Ave ± SD) | 1.82 ± 1.06 | 1.95 ± 1.11 | 1.54 ± 1.00 | 2.02 ± 1.01 | 1.69 ± 1.08 |
|  |  |  |  |  |  |
| *: p<0.05 between NDH and NDC, **: p<0.05 between NDH and DC  BMI: body mass index, RAS: renin angiotensin system, NDH: non-dipper high, NDC: non-dipper controlled, DC: dipper controlled | | | | | |

Supplementary Table 3. Patient characteristics of each group with eGFR ≥30 ml/min/1.73m^2^

|  | Total | Dipper high | Dipper controlled | Non-dipper high | Non-dipper controlled |
| --- | --- | --- | --- | --- | --- |
| Number | 578 | 33 | 52 | 286 | 207 |
| Men (%) | 67.5 | 69.7 | 59.6 | 72.7 | 61.8 |
| Age (years old) | 72 (64 - 79) | 71 (59 - 78) | 71 (64 - 81) | 70 (63 - 78) | 73 (65 - 80) |
| BMI (kg/m^2^) | 23.18 (20.85 - 25.64) | 24.08 (21.13 - 27.48) | 23.15 (20.75 - 24.96) | 23.66 (21.44 - 26.15) | 22.77 (20.22 - 24.94) |
| Diabetes mellitus (%) | 42.7 | 57.6 | 26.9 ** **** | 51.1 | 32.9 * |
| Creatinine (mg/dL) | 2.67 (2.15 - 3.67) | 3.02 (2.43 - 4.22) | 2.45 (2.01 - 3.46) | 2.88 (2.25 - 4.04) | 2.44 (2.07 - 3.26) * |
| eGFR (ml/min/1.73m^2^) | 19.69 (13.85 - 24.92) | 18.73 (11.24 - 22.01) | 21.12 (14.58 - 26.73) | 18.16 (12.57 - 23.81) | 21.39 (15.76 - 25.41) * |
| Blood urea nitrogen (mg/dL) | 39.90 (31.40 - 52.70) | 43.60 (34.80 - 61.20) | 35.80 (29.98 - 51.03) | 41.00 (30.73 - 51.68) | 39.30 (32.35 - 51.35) |
| Urinary protein (g/day) | 0.74 (0.17 - 2.29) | 0.97 (0.31 - 2.15) | 0.26 (0.13 - 1.00) ** | 1.56 (0.59 - 3.95) | 0.30 (0.06 - 0.82) * *** |
| Hemoglobin (g/dL) | 10.40 (9.13 - 11.70) | 10.50 (9.00 - 12.10) | 10.40 (9.18 - 12.30) | 10.30 (9.20 - 11.50) | 10.50 (9.10 - 11.70) |
| Calcium channel blockers (%) | 75.1 | 90.9 | 67.3 | 81.8 | 65.2 * *** |
| RAS inhibitors (%) | 76.5 | 75.8 | 63.5 | 79.0 | 76.3 |
| Diuretics (%) | 41.2 | 45.5 | 28.9 | 44.8 | 38.7 |
| β-blockers (%) | 17.5 | 15.2 | 19.2 | 19.2 | 15.0 |
| Number of antihypertensive drug classes (Ave ± SD) | 2.10 ± 1.00 | 2.27 ± 0.88 | 1.79 ± 1.13 | 2.25 ± 0.96 | 1.95 ± 1.01 |
|  |  |  |  |  |  |
| *: p<0.05 between NDH and NDC, **: p<0.05 between NDH and DC, ***: p<0.05 between NDC and DH, ****: p<0.05 between DC and DH  BMI: body mass index, RAS: renin angiotensin system, NDH: non-dipper high, NDC: non-dipper controlled, DH: dipper high, DC: dipper controlled | | | | | |

Supplementary Table 4. Patient characteristics of each group with eGFR <30 ml/min/1.73m^2^

|  | Total | Dipper high | Dipper controlled | Non-dipper high | Non-dipper controlled |
| --- | --- | --- | --- | --- | --- |
| 24-hour (mmHg) |  |  |  |  |  |
| Systolic BP | 126 (116 - 137) | 134 (131 - 143) | 116 (111 - 123) * | 139 (134 - 147)§ | 119 (111 - 124) †‡ |
| Diastolic BP | 76 (69 - 81) | 77 (75 - 83) | 73 (67 - 76) * | 81 (75 - 88)§ | 72 (66 - 77) †‡ |
|  |  |  |  |  |  |
| Daytime (mmHg) |  |  |  |  |  |
| Systolic BP | 128 (119 - 138) | 144 (139 - 151) | 124 (118 - 130) * | 139 (134 - 147) **§ | 120 (112 - 125) †‡ |
| Diastolic BP | 77 (71 - 84) | 83 (79 - 88) | 77 (71 - 81) * | 82 (76 - 90)§ | 73 (68 - 79) ‡ |
|  |  |  |  |  |  |
| Nighttime (mmHg) |  |  |  |  |  |
| Systolic BP | 124 (113 - 137) | 123 (121 - 132) | 108 (101 - 112) *♯ | 139 (134 - 148) **§ | 117 (110 - 123) †‡ |
| Diastolic BP | 73 (66 - 80) | 72 (70 - 76) | 64 (59 - 70) *♯ | 81 (74 - 88) **§ | 70 (65 - 75) † |
|  |  |  |  |  |  |
| BP dipping (%) | 2.6 (-2.2 - 7.0) | 13.8 (12.2 - 14.8) | 12.7 (11.3 - 15.0) *♯ | 0.0 (-3.9 - 4.7)§ | 2.4 (-1.6 - 5.9) †‡ |
|  |  |  |  |  |  |
| *: p<0.005 between NDH and DC, **: p<0.005 between NDH and DH, ♯: p<0.005 between NDC and DC,  †: p<0.005 between DC and DH, ‡: p<0.005 between NDC and DH, §:p<0.005 between NDC and NDH  BP: blood pressure, NDH: non-dipper high, NDC: non-dipper controlled, DH: dipper high, DC: dipper controlled | | | | | |

Supplementary Table 5. Average BP profiles of each group with eGFR ≥30 ml/min/1.73m^2^.

|  | Total | Dipper high | Dipper controlled | Non-dipper high | Non-dipper controlled |
| --- | --- | --- | --- | --- | --- |
| 24-hour (mmHg) |  |  |  |  |  |
| Systolic BP | 132 (120 - 146) | 143 (138 - 151) | 116 (111 - 124) * | 144 (136 - 153)§ | 118 (111 - 124) †‡ |
| Diastolic BP | 76 (69 - 84) | 81 (71 - 89) | 71 (66 - 77) * | 81 (76 - 88)§ | 70 (65 - 76) †‡ |
|  |  |  |  |  |  |
| Daytime (mmHg) |  |  |  |  |  |
| Systolic BP | 133 (121 - 147) | 151 (145 - 161) | 123 (117 - 131) *♯ | 144 (136 - 154) § | 119 (112 - 125) †‡ |
| Diastolic BP | 77 (71 - 85) | 85 (75 - 96) | 75 (71 - 82) * | 82 (76 - 90)§ | 71 (67 - 77) ‡ |
|  |  |  |  |  |  |
| Nighttime (mmHg) |  |  |  |  |  |
| Systolic BP | 131 (116 - 145) | 133 (125 - 139) | 107 (103 - 115) *♯ | 144 (136 - 155) **§ | 117 (110 - 124) †‡ |
| Diastolic BP | 74 (67 - 83) | 75 (67 - 82) | 65 (58 - 69) *♯ | 81 (74 - 88) **§ | 69 (63 - 75) † |
|  |  |  |  |  |  |
| BP dipping (%) | 3.0 (-2.8 - 7.1) | 12.5 (11.7 - 13.8) | 12.8 (11.2 - 16.0) ♯ | 0.2 (-4.5 - 4.9)§ | 2.3 (-2.7 - 5.7) †‡ |
|  |  |  |  |  |  |
| *: p<0.005 between NDH and DC, **: p<0.005 between NDH and DH, ♯: p<0.005 between NDC and DC,  †: p<0.005 between DC and DH, ‡: p<0.005 between NDC and DH, §:p<0.005 between NDC and NDH  BP: blood pressure, NDH: non-dipper high, NDC: non-dipper controlled, DH: dipper high, DC: dipper controlled | | | | | |

Supplementary Table 6. Average BP profiles of each group with eGFR <30 ml/min/1.73m^2^.

Supplementary Table 7. Detailed profiles of the annual incidence of loss of follow-up, including the incidence of outcomes, stopping of visits, and death from non-renal causes in the patients with eGFR ≥30 ml/min/1.73m^2^.

Supplementary Table 8. Detailed profiles of the annual incidence of loss of follow-up, including the incidence of outcomes, stopping of visits, and death from non-renal causes in the patients with eGFR <30 ml/min/1.73m^2^.


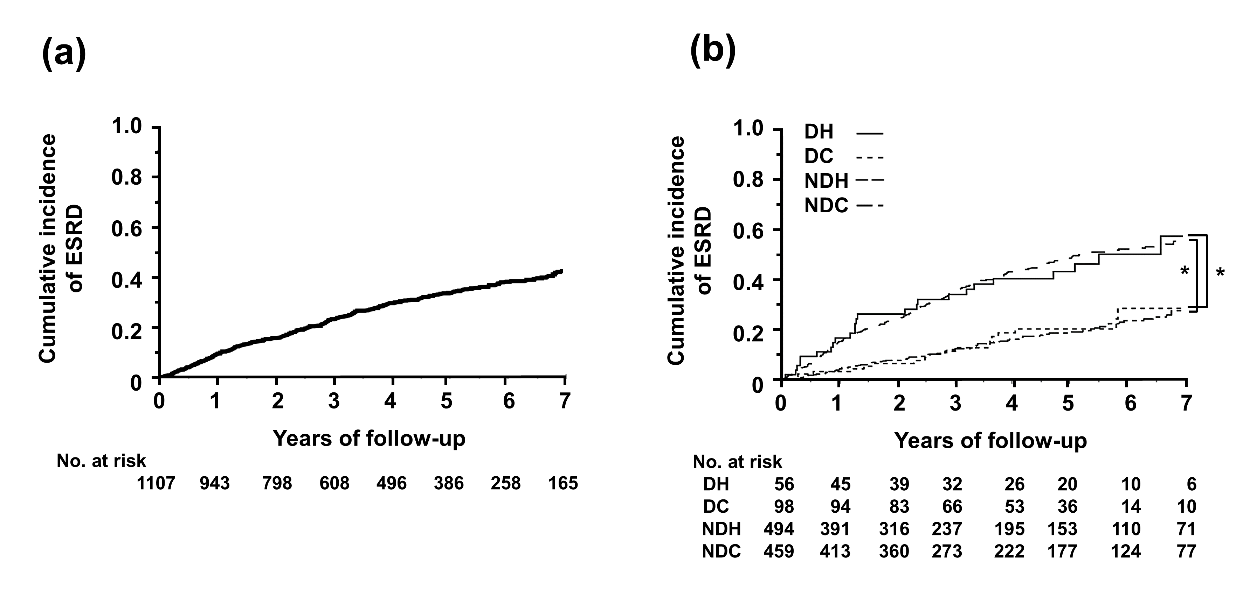


Supplementary Figure 1. Kaplan-Meier plots of the cumulative incidence of ESRD.

(a) Among all patients, the cumulative incidence rates of ESRD at 1, 3, and 7 years after recruitment were 9.2, 22.1, and 39.4%, respectively.

(b) The cumulative incidence of ESRD was greater in patients with hypertension than in those with normotension. *: Wilcoxon’s test p < 0.05.


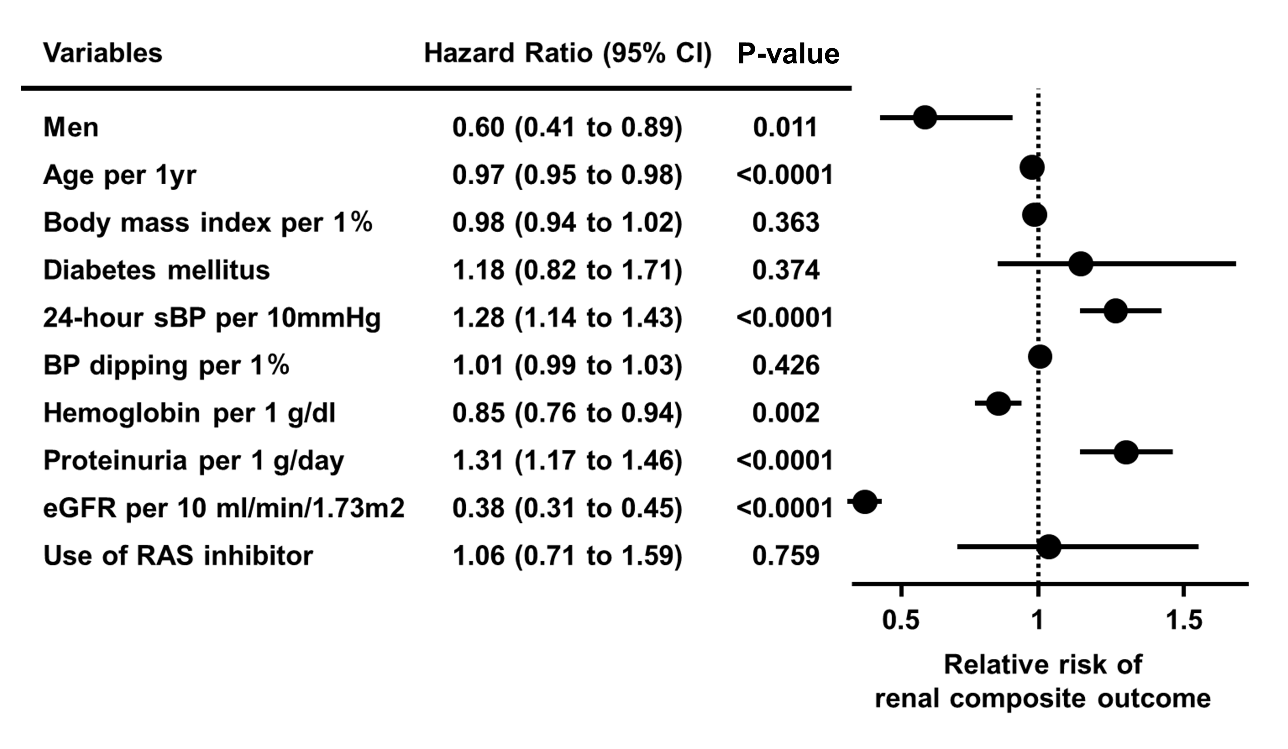


Supplementary Figure 2. Cox regression analysis for the identification of factors associated with the incidence of ESRD. Filled circles represent the hazard ratio and horizontal lines denote the 95% confidence intervals (95% CI).


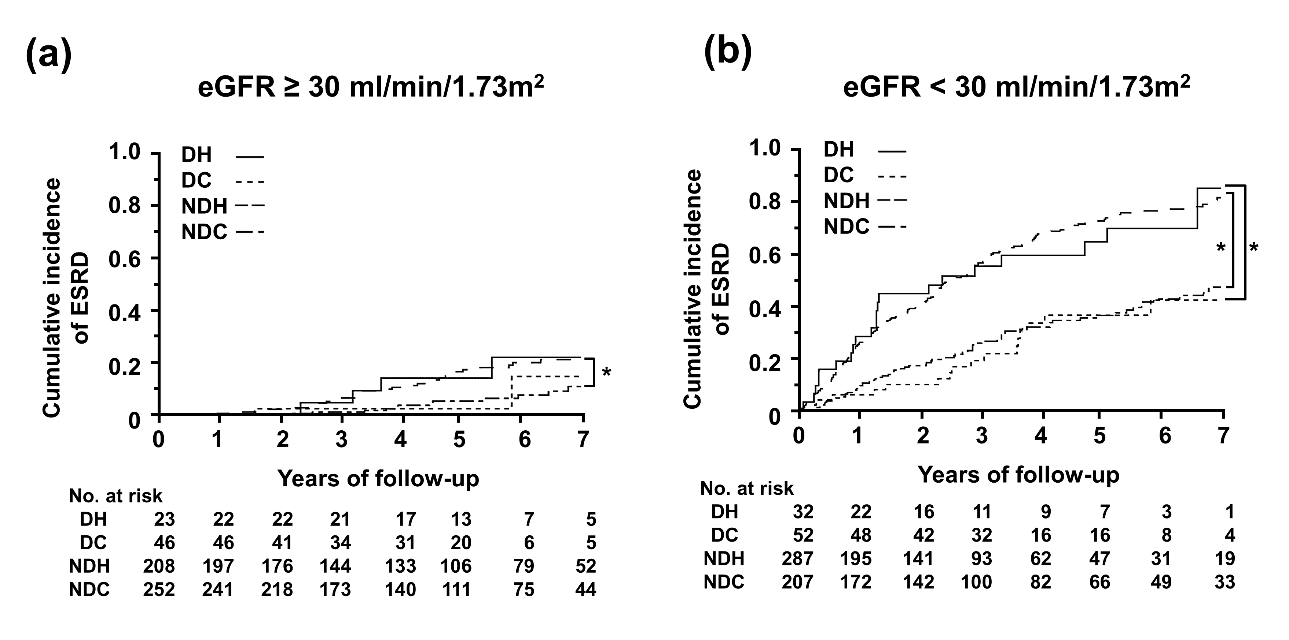


Supplementary Figure 3. Kaplan-Meier plots of a separate analysis of the cumulative incidence of ESRD in early and advanced CKD patients.

In early (a) and advanced (b) CKD patients, the cumulative incidence of ESRD was greater in patients with hypertension than in those with normotension. *: Wilcoxon’s test p < 0.05
